# Cytomegalovirus meningoencephalitis in an immunocompetent host with evolution of cystic encephalomalacia

**DOI:** 10.1016/j.radcr.2025.04.053

**Published:** 2025-05-12

**Authors:** Derryl J Miller, Jade Davis, Whitney Gervelis, Saurabh Singhal, Susan Conrad

**Affiliations:** a Department of Neurology, Indiana University School of Medicine, Indianapolis, IN, USA; b Department of Child Neurology, South Bend Child Neurology, South Bend, IN, USA; c Department of Child Neurology, Gundersen Lutheran Medical Center, La Crosse, WI, USA; d Department of Neurology, WellSmart Health Neurology Clinic, Opelousas, LA, USA

**Keywords:** Meningoencephalitis, Cytomegalovirus, Cystic encephalomalacia, Magnetic resonance imaging, Computed tomography, Drug resistant epilepsy

## Abstract

A previously healthy 2-month-old male presented with fever and seizures. Cerebrospinal fluid (CSF) studies, head computed tomography (CT), and brain magnetic resonance imaging (MRI) studies were unremarkable on admission. After a clinical decline on the second day admission due to cerebral edema a repeat head CT showed loss of gray-white matter differentiation indicative of global anoxic injury. A repeat brain MRI on day 5 revealed diffuse restricted diffusion globally, acute infarction of the right posterior inferior cerebellar artery territory, and left frontoparietal leptomeningitis versus cortical laminar necrosis. A repeat LP was done on day 5 and showed a total nucleated cell count of 42 cells/mm^3 with 39% lymphocytes and 61% monocytes, glucose 52 mg/dL. He made steady clinical improvement with supportive care. CMV was detected on the repeat CSF sample using PCR on day 11 and he was started on ganciclovir for CMV meningoencephalitis. CMV should be considered as an etiology for viral meningoencephalitis even in immunocompetent patients. Our case also shows the classic imaging evolution of CMV meningoencephalitis.

## Introduction

Cytomegalovirus (CMV) is known to cause severe illness in immunocompromised individuals and those infected congenitally. In the majority of immunocompetent patients, CMV infection is asymptomatic or associated with a mononucleosis-like illness, due to a cell mediated immune response. There are a growing number of case reports demonstrating CMV infection in immunocompetent patients manifesting as colitis, meningoencephalitis, venous or arterial thrombosis, uveitis, and pneumonitis [1]. We present a case of CMV meningoencephalitis in an immunocompetent infant complicated by diffuse anoxic brain injury, ischemic stroke, seizures, and marked cerebral volume loss.

The clinical presentation of CMV meningoencephalitis is characterized by fever, headache, altered mental status, seizures, and focal neurologic signs. Diagnosis is based on detection of CMV virus in cerebrospinal fluid (CSF) by polymerase chain reaction (PCR) or through detection of intrathecal serologies. PCR has a very high sensitivity and specificity for the CSF of 86%-100% [4].

### Case

A previously healthy 2-month-old boy presented with subjective fever and suspected seizures. He was previously born via spontaneous vaginal delivery at term after an uncomplicated pregnancy and received his first hepatitis B vaccination. He had not yet received his 2-month vaccinations due to age at the time of presentation. He was developing without concerns, though had mild plagiocephaly.

A day before presentation he developed a tactile temperature that seemed to improve with acetaminophen and then demonstrated focal impaired awareness seizures with eyes deviating to the right and twitching of the right more than left, which prompted his caregiver to call emergency medical services. At an outside hospital, he was started on ceftriaxone, ampicillin, azithromycin and acyclovir, intubated and loaded with fosphenytoin and phenobarbital intravenously. There was concern for status epilepticus, so he was transported Riley Hospital for Children with anesthetic coma with midazolam drip for monitoring on continuous electroencephalography (EEG) for seizures.

On admission to our institution, he was intubated, sedated, and his temperature was 36.5°C with otherwise stable vital signs. His right pupil was larger than the left but reactive. There was no nystagmus and eyes were conjugate. Face was symmetric. Muscle tone was increased equally in all extremities. Patellar reflexes were symmetrically brisk with clonus bilaterally. The remainder of the physical exam was unremarkable with the exception of periorbital edema and a relatively large, nonbulging anterior fontanel.

**Fig. 1:**
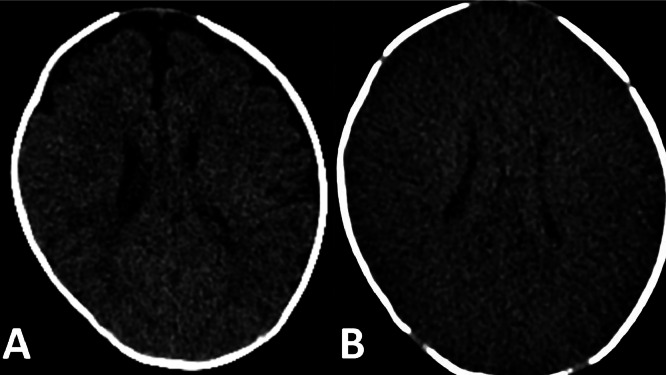
(A) Axial computed tomography (CT) of the head without contrast on day 1 which was read as normal at the outside hospital. (B) Axial CT of the head without contrast on day 2 after clinical changes were noted with diffuse loss of grey-white differentiation and sulcal effacement.

**Fig. 2:**
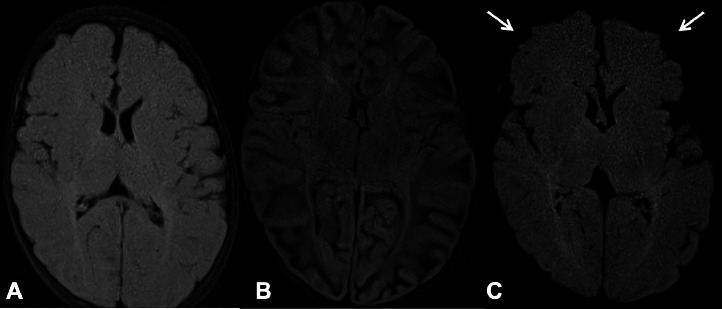
(A) Axial fluid attenuated inversion recovery (FLAIR) Magnetic Resonance Imaging (MRI) of the brain without contrast from outside hospital on day of presentation which was read as normal. (B) Axial Diffusion Weighted Imaging (DWI) MRI of the brain without contrast on day 5 showing lesions in the bilateral cerebral cortices, corpus callosum, and deep gray nuclei, consistent with diffuse anoxic brain injury. (C) Axial FLAIR MRI of the brain with hyperintensities involving the frontoparietal cortices with mild brain volume loss with white arrows highlighting areas of encephalomalacia.

Laboratory investigations included a white blood cell count (WBC) of 14000/μl with no bandemia, sodium 133 mEq/L, ALT 70 U/L, AST 67 U/L alkaline phosphatase 364 U/L, and ammonia 64 mcg/dL. Electrolytes were within normal limits and a cerebrospinal fluid meningoencephalitis PCR panel including CMV and herpes simplex virus (HSV) was negative. CSF studies showed total nucleated cell (TNC) count of 6 cells/mm^3^, Red Blood Cell (RBC) 2 cells/mm^3^, glucose 54 mg/dL, protein 87 mg/dL. Blood culture was negative and CSF culture and stain were negative. Head CT without contrast and MRI of the brain without contrast from the outside hospital were unremarkable, but, on day 2 of presentation, he was found to have a fixed and dilated pupil on exam and a head CT without contrast showed loss of gray-white matter differentiation indicative of global anoxic injury (Fig. 1). This clinical change was believed to be due to cerebral edema causing an occulomotor nerve palsy due to compression [5]. He continued to have intermittent seizures despite maintenance phenobarbital and fosphenytoin during the first few days of his course. He was monitored on continuous electroencephalography (EEG) during this time and developed periodic lateralized epileptiform discharges (PLEDS) localized maximally in the left frontocentral head region with multifocal epileptiform discharges in the background and generalized encephalopathy. He received additional loads of fosphenytoin and levetiracetam while the midazolam drip was weaned. He was continued on vancomycin, ceftriaxone and acyclovir until the blood and CSF cultures were negative at 48 hours at which time empiric therapies were stopped. Work up for an underlying metabolic disorder was unrevealing including homocystine, vitamin B12, urine orotic acid, serum amino acids, acetyl carnitine, carnitine, and CK. He had a slightly elevated methylmalonic acid which was attributed to low maternal vitamin B12. A repeat brain MRI on day 5 revealed diffuse anoxic brain injury, acute infarction of the right posterior inferior cerebellar artery (PICA) territory, and left frontoparietal leptomeningitis versus cortical laminar necrosis (Fig. 2). A repeat LP was done at 5 days that showed a TNC count of 42 cells/mm^3 with 39% lymphocytes and 61% monocytes. He developed a right ptosis with eye edema and a Magnetic Resonance Venography (MRV) of the head was ordered but did not reveal any central venous thrombosis. Ophthalmology was consulted and determined that the right eye anisocoria and ptosis was likely due to the PICA infarct causing a right cranial nerve (CN) III palsy. While infectious etiology was strongly suspected given the rise in TNC count in CSF, a nonaccidental trauma work-up was also conducted due to the concern for the global anoxic injury seen on imaging. There was no history of trauma, and there were no retinal hemorrhages seen on retinal exam, no external injuries, and no injuries on skeletal X-ray survey.

Despite the negative prognostic indicators on imaging and no clear etiology the patient made steady clinical improvement daily along with cessation of seizures. He was extubated on day 7 and transferred to the inpatient floor. A repeat meningoencephalitis PCR panel came back positive for cytomegalovirus (CMV) on day 11, and he was subsequently started on ganciclovir. A blood CMV PCR was also positive. When discharged home from the hospital, the infant was feeding all by mouth, moving all extremities, but did have problems with visual tracking which he did not have prior to the illness.

Work up for an underlying immunodeficiency included HIV testing and T and B cell subsets which were unremarkable. He was not found to have any signs of congenital CMV infection such as chorioretinitis, periventricular calcifications, or hearing loss. He completed a treatment course of ganciclovir. On follow up, this patient’s quantitative CMV PCR levels were decreasing.

**Fig. 3:**
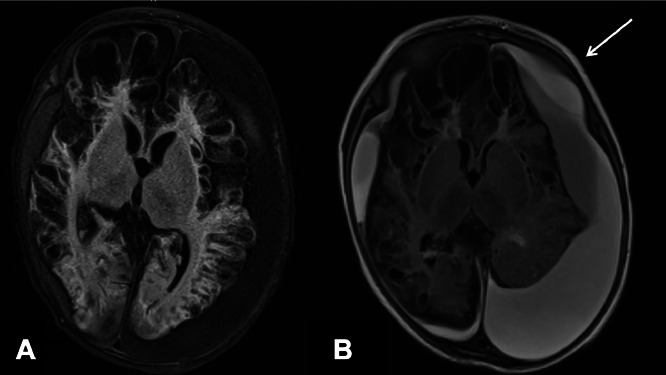
(A) Repeat axial FLAIR MRI of the brain without contrast at 22 days showing diffuse frontoparietotemporal predominant encephalomalacia. (B) Repeat axial FLAIR MRI of the brain without contrast at 2 months after presentation with diffuse severe cystic encephalomalacia of the entire brain with white arrow indicating more severe encephalomalacia in the left hemisphere than the right with associated subdural fluid collections.

Follow up MRI studies of the brain without contrast at 22 days and 2 months showed significant atrophy and cortical injury which is maximal in the periventricular head regions consistent with CMV (Fig. 3).

Following discharge, he is followed by our group. At 8 years old, he has spastic quadriparesis which is right more than left with positive Babinski sign on the right. He also has drug resistant focal epilepsy with tonic seizures and myoclonus. He has global developmental delay. He does not sit without assistance, does not ambulate independently, and does not speak other than for babbling. His seizures are treated with Levetiracetam, Clobazam, Valproic Acid, and the ketogenic diet but are ongoing of daily to every other day with brief tonic seizures and myoclonus.

## Discussion

Presented here is the case of an immunocompetent 2-month-old who presented with fever and seizures due to postnatally acquired CMV meningoencephalitis. The diagnosis is made by positive CMV PCR from CSF and supported by a positive serum CMV PCR test. CMV serologies are also utilized to support this diagnosis along with urine CMV studies. Postnatal CMV acquisition can occur by transmission via infected breast milk from mother or contact with vaginal secretions at the time of birth. Most term infants do not develop clinical illness due to the presence of maternal CMV antibodies transferred in the milk.

Most patients with CMV infection have history of solid organ transplantation, immunocompromise from human immunodeficiency virus, in patients with sensorineural hearing loss, and polymorphisms in Toll-Like Receprors or Mannose-Binding Lectin genes [6]. While severe CMV infections in immunocompetent patients are rare, there are other cases reported in the literature [1]. As the utilization of PCR testing on CSF increases, we may continue to diagnose CMV CNS infections in immunocompetent patients. A systematic review of CMV infections suggests CMV most commonly affects the gastrointestinal tract and central nervous system [1]. CMV infections are also associated with thrombosis, although not specifically in the central nervous system [1]. Genetic susceptibility to common pathogens, such as herpes simplex virus (HSV), in otherwise healthy patients has been recorded and may have played a role in this case [2].

The choice and duration of antiviral therapy for postnatally acquired CMV have not been standardized [3]. The most common regimen included initiation with intravenous ganciclovir with transition to oral valganciclovir with an average duration of 6-7 weeks [3]. Response to treatment is monitored with quantitative serum CMV PCR testing to detect decrease in viral load. Interestingly, there are case reports of CMV CNS infection in immunocompetent patients in which symptoms resolved without CMV-specific antiviral treatment and our own patient’s condition was improving before he received any treatment, though with obvious severe injury to his brain on imaging studies [4].

The outcomes of other immunocompetent patients in the literature are varied. Rhardjo et al. reported a case of an adult male who had severe disease with brainstem findings due to CMV meningoencephalitis who slowly recovered after initiation of ganciclovir treatment [7]. Our patient made clinical improvement and eventually was discharged home on oral ganciclovir treatment, although repeat brain MRI before discharge showed cystic encephalomalacia with diffuse cerebral volume loss, indicating a high possibility of long-term sequelae, including spastic quadriparesis, cortical blindness, focal epilepsy, and developmental delay.

His global anoxic injury may have been caused from prolonged seizure activity. Prolonged seizure activity and status epilepticus causes neuronal damage [8,9]. There was reported seizure activity at home prior to his presentation to the outside hospital and continuous EEG monitoring was not initiated until after transfer, which may have resulted in unrecognized subclinical status epilepticus during the initial presentation. The initial head imaging showed no evidence of injury at presentation, though this finding may have been delayed because of delayed cell death from apoptosis [10].

## Conclusion

We present an unusual CMV meningoencephalitis infection in an otherwise immunocompetent patient which resulted in severe brain injury with encephalomalacia noted on serial imaging studies. Although the patient did make a gradual recovery, the injury that was suffered remains severe and will impact this patient developmentally. In addition, the patient also suffered from other co-morbidities during his illness, including prolonged seizures, CN III palsy, global anoxic injury, and ischemic stroke. This case also illustrates the value of repeat lumbar punctures if a patient’s symptoms are suspected to be infectious in nature with bland initial CSF studies. CMV should be incorporated into the differential when caring for a patient with suspected viral meningoencephalitis even when there is no underlying immunodeficiency.

## Ethical statement

We confirm that we have read the Journal’s position on issues involved in ethical publication and affirm that this report is consistent with those guidelines.

## Patient consent

Written consent was acquired from the family prior to drafting this manuscript.
